# Zein-based nano-delivery systems for encapsulation and protection of hydrophobic bioactives: A review

**DOI:** 10.3389/fnut.2022.999373

**Published:** 2022-09-28

**Authors:** Xiaojia Yan, Moting Li, Xingfeng Xu, Xuebo Liu, Fuguo Liu

**Affiliations:** ^1^College of Food Science and Engineering, Northwest A&F University, Xianyang, China; ^2^College of Food Science and Engineering, Qingdao Agricultural University, Qingdao, China

**Keywords:** zein, self-assembly, nanoparticle, delivery, curcumin

## Abstract

Zein is a kind of excellent carrier materials to construct nano-sized delivery systems for hydrophobic bioactives, owing to its unique interfacial behavior, such as self-assembly and packing into nanoparticles. In this article, the chemical basis and preparation methods of zein nanoparticles are firstly reviewed, including chemical crosslinking, emulsification/solvent evaporation, antisolvent, pH-driven method, etc., as well as the pros and cons of different preparation methods. Various strategies to improve their physicochemical properties are then summarized. Lastly, the encapsulation and protection effects of zein-based nano-sized delivery systems (e.g., nanoparticles, nanofibers, nanomicelles and nanogels) are discussed, using curcumin as a model bioactive ingredient. This review will provide guidance for the in-depth development of hydrophobic bioactives formulations and improve the application value of zein in the food industry.

## Graphical Abstract

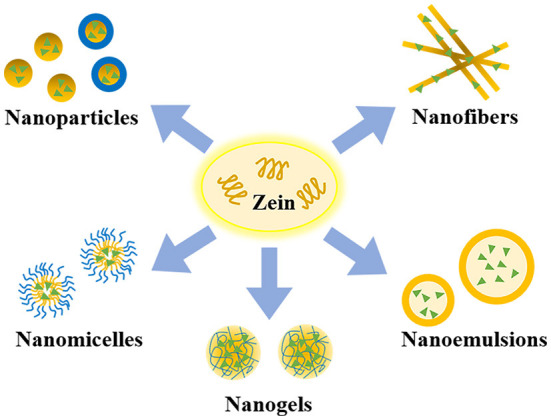

## Introduction

**Figure 1:**
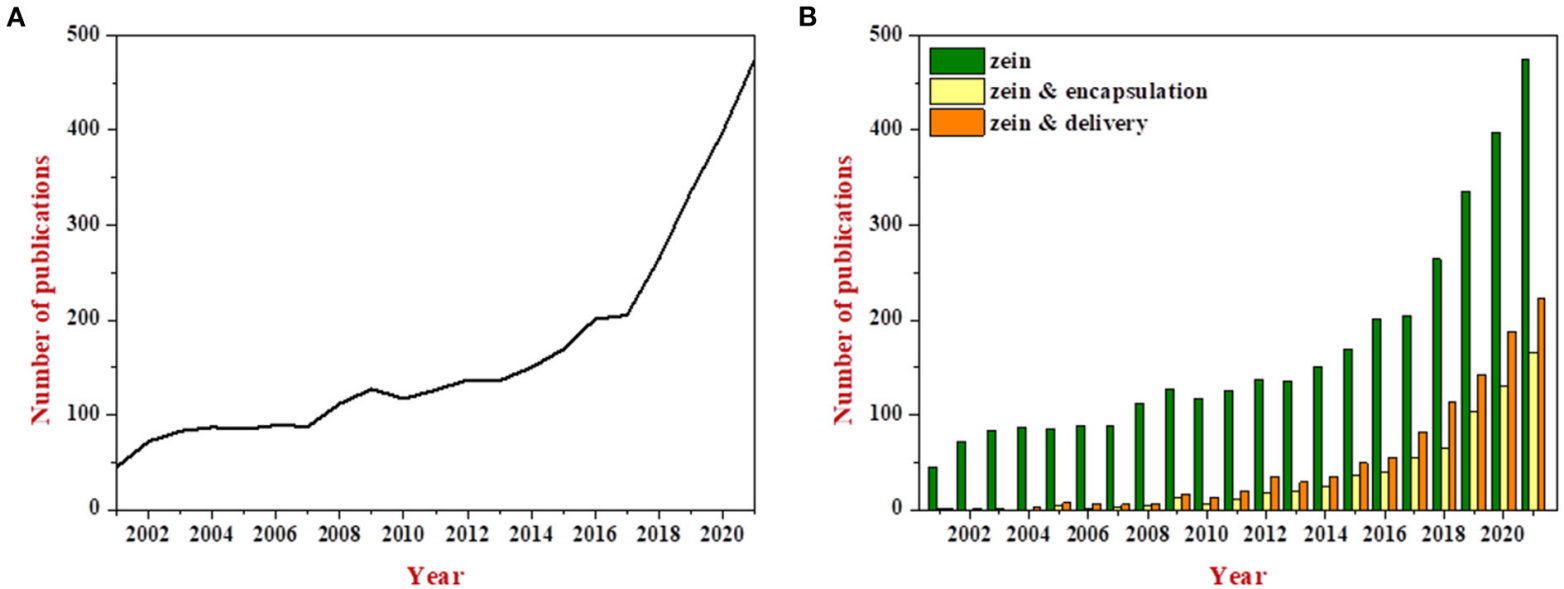
Evolution of number of publications related to the term “zein” during the years 2001 to 2021 **(A)**; Comparison of the number of publications related to the terms “zein” vs. “zein & encapsulation” and “zein & delivery” **(B)**. All the data were obtained through the Web of Science system.

Zein is the main storage protein in maize and accounts for 30%–60% of total protein content (1). It contains more than 50% of non-polar amino acids, including leucine, proline, alanine, phenylalanine, valine and isoleucine, but lacks basic and acidic amino acids (2). The unbalance of amino acid composition leads to the low nutritional value of zein. However, zein readily self-assembles into various structures depending on the solvents and the processing conditions, and it has been extensively studied in terms of encapsulating and delivering bioactive ingredients, which can be well–reflected in the yearly increase in number of publications related to the terms “zein & encapsulation” and “zein & delivery”, as shown in Figure 1. About half of the publications on zein are related to research for encapsulation and delivery over the past 3 years.

In the past two decades, researchers have successively proposed that the construction of nano-sized delivery systems to encapsulate active ingredients can maximize their health effects, which is mainly attributed to (i) the high specific surface area of nanocarriers can increase the solubility and dissolution rate of actives; (ii) its nanoscale size enables the actives to circulate in the system for a longer time, improves actives distribution in the body as well as allows targeted release and increases biofilm permeability (3). Based on this, the solubility and bioavailability of hydrophobic active ingredients can be effectively enhanced with the help of nanocarriers. Common nano-sized delivery systems include nanoparticles, nanofibers, nanomicelles, solid lipid nanoparticles and nanoemulsion. Biopolymers are alternative materials for constructing nanocarriers because of their good biocompatibility, and Zein-based nano-sized delivery systems loaded with hydrophobic active ingredients have been constructed. For example, maytansine-loaded zein nanoparticles enhanced tumor targeting for non-small cell lung cancer (A549 cells) and reduced the toxic side effects of maytansine (4). Curcumin-loaded zein nanofibers showed enhanced antibacterial activity and the inhibition efficiency increased with the increase of curcumin content (5). Dispersibility of curcumin encapsulated in whey protein isolate (WPI)-zein composite nanoparticles was significantly improved (6). Zein-hyaluronic acid composite nanoparticles prepared through layer-by-layer assembly technique effectively improved the light, thermal and storage stability of curcumin and quercetagetin (7). With the help of nano-delivery system, the water solubility and chemical stability of encapsulated lutein were significantly improved, and the bioaccessibility (32.11%) was much higher than that of free lutein (16.21%) (8). In addition, many studies on hydrophobic active ingredients, such as quercetin, resveratrol, rutin, β-carotene, Vitamin D3, retinol encapsulated in zein-based nanocarriers have been reported (9–14).

In this review, we mainly introduce the chemical basis of zein and various preparation methods of zein nanoparticles, and summarize recent reports on zein-based composite nanoparticles, using curcumin as a model bioactive ingredient. In addition to zein nanoparticles, other types of zein delivery systems are also discussed, including nanofiber, nanomicelles, etc.

## Chemical basis of zein

### Composition and general properties

According to the difference in molecular weight and solubility, zein can be divided into four categories: α-, β-, γ-, δ-zein. Among them, α-zein accounts for 70%–85% of the total zein content, with a molecular weight of 19–22 kDa, and it is soluble in 70%–95% ethanol aqueous solution. β-zein is composed of α-zein linked by disulfide bonds, so its molecular weight is high. It is soluble in 60%–90% ethanol aqueous solution (15). Generally, Except for a certain concentration of alcohol solution, zein can also be dissolved in alkaline solutions with pH ≥ 11.5 (16, 17), and solutions with high concentration urea and other reducing agents (18). Although zein has poor water solubility, unbalanced amino acid composition and low nutritional value, its unique solubility makes it easy to self-assemble into zein micro/nanoparticles. Therefore, zein is an ideal carrier material for hydrophobic bioactive ingredients. It is also more anti-digestive than other proteins, which is one of the advantages as an oral delivery carrier.

### Molecular structure

The self-assembling mechanism of zein is closely related to its structure. Several molecular models for the tertiary structure of α-zein have been proposed, including cylinder model, ribbon-like model, hairpin model and superhelical structural model (19).

**Figure 2:**
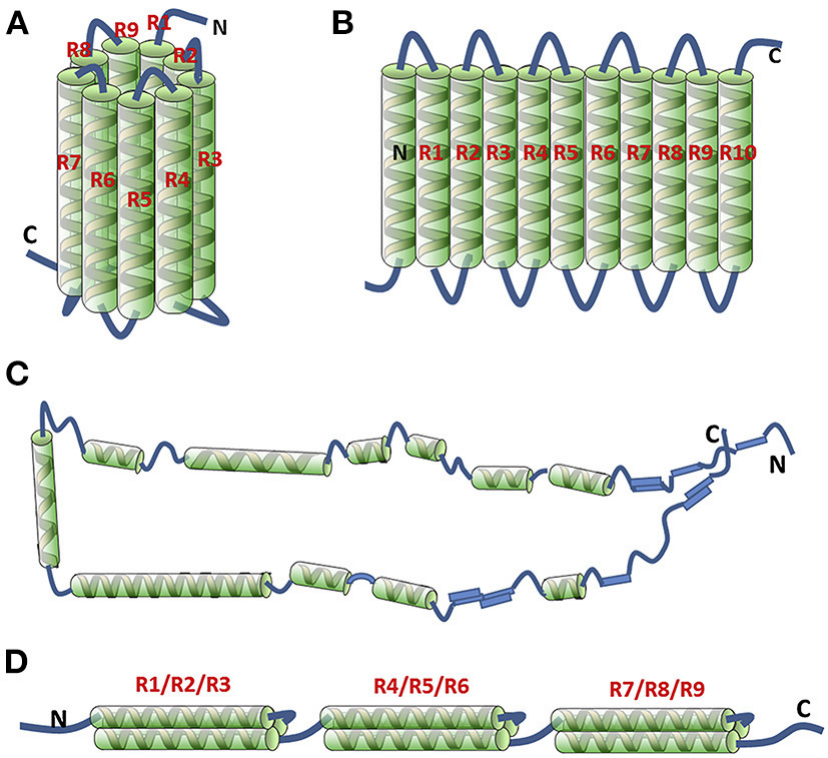
Proposed 3-D structural models of α-zein. Cylindrical model **(A)**; ribbon-like model (22 kDa) **(B)**; hairpin model (19 kDa) **(C)**; super helical structural model (19 kDa) **(D)**. “R” means repeat unit. Reprinted from (19) with permission from Elsevier.

The cylinder model is based on analysis of the repeat sequence units and α-helix content of zein dissolved in 70% methanol aqueous solution (20). There are 50%–60% α-helices in zein molecules, and the rest are turn and random coils. Each primary structure of a zein molecule consists of 20 amino acid residues repeated 9 times. Analysis of the physical properties (hydration potential, polarity, turn, helix formation tendency) indicates that the amino acid sequence containing repeat fragments consists of α-helixes with turn regions on both sides. The helix regions are composed of numerous polar amino acids and several hydrophobic amino acid residues, and the turn regions are rich in glutamine. In the structural model of zein, nine adjacent, topologically antiparallel homologous helices cluster form a cylindrical surface connected by polar glutamine residues (Figure 2A). The structure is stabilized by van der Waals force and intramolecular and intermolecular hydrogen bonds. Glutamine-rich turns are located between the helical structures and the cylindrical cap, facilitating the intermolecular stacking into a plane by the interaction between the side chains. The structure model can explain the dense, membrane-enveloped deposits formed by proteins in maize seeds.

To modify the cylinder model, the structure of zein dissolved in 70% ethanol aqueous solution was further analyzed by small angle X-ray scattering (SAXS) (21). In the ribbon-like model, zein is composed of 9–10 adjacent, topologically antiparallel homologous helices clustered, presenting a slender rectangular prism structure with an axial ratio of 6:1, and its three dimensions are 13, 3 and 1.2 nm (Figure 2B). The two-dimensional intermolecular hydrophobic aggregation makes the side chains in the helixes accumulate into a hydrophobic surface, forming a structure with hydrophilic ends and hydrophobic sides, which greatly improves the structural stability of α-zein. The two-dimensional ribbon-like model has been widely used to explain the different orientations of zein molecules on hydrophilic/hydrophobic surfaces and the self-assembly of zein from single molecule to nanoparticles.

In addition, the hairpin model based on nuclear magnetism resonance (NMR), SAXS and Fourier transform infrared spectroscopy (FTIR) is also composed of a series of helix structures as shown in Figure 2C (22). Different from the above two models, these helix structures are connected and extended in the form of rings, sheets or loops, so zein can fold or extend in other ways in different solvent environments. At the same time, it is thought to be related to the fibrillation ability of zein.

In 2006, a model consisting of three superhelix structures was built by using three antiparallel helix segments connected by a glutamine-rich turn, as shown in Figure 2D (23).

Despite the differences in the molecular models above, it is generally believed that α-zein is a slender conformation formed by a large number of α-helix structures. The three-dimensional structure of zein is affected by different factors, especially solvent type (24). Compared with the folded structure model, the slightly larger sizes of α-zein in acetic acid, 70% and 80% ethanol aqueous solutions indicate that α-zein structure is locally unfolded in these solutions. The CD data demonstrate the secondary structure of α-zein do not change significantly, either in acetic acid or in 70% and 80% ethanol aqueous solutions, which may be because local unfolding mostly occurs in the coil region. The size of α-zein in acetic acid is larger than that in ethanol aqueous solutions, indicating that the expansion and swelling degree of α-zein is greater in the presence of acid. With the increase of zein concentration, α-zein dissolved in acetic acid behaves like polyelectrolyte dissolved in an excellent solvent, and its particle size decreases slightly. Moreover, α-zein dissolved in ethanol aqueous solutions behaves like polymer dissolved in θ solvent (alcohol solution). The conformation and dissolution behaviors of α-zein both differ among different solvents, which may be attributed to three causes: (i) According to the similarity-intermiscibility theory, the dissolution behavior of α-zein should be related to the polarity of solvent. It is known that the hydrophilic index of water, acetic acid and ethanol is 9.0, 6.2, and 5.2 respectively referring to the solvent miscibility table, indicating the hydrophilic index is very close between acetic acid and 70% or 80% ethanol aqueous solutions. The hydrophilic index of α-zein with a molecular weight of 19 and 22 KDa is 4.70 and 4.80 respectively (25), so the similarity-intermiscibility theory should not be the main reason accounting for the difference of solvent quality. (ii) The differences can be related to the hydrophilicity/hydrophobicity of the protein surface. Partial unfolding of zein may change the solvent accessible area of zein surface and hydration. As local unfolding of α-zein is severer in acetic acid, the solvent accessible area is larger, thus promoting the dissolution of α-zein. (iii) Protein surface protonation can also inhibit protein aggregation. Protonation of zein reduces hydrophobicity and improves stability of zein solution through the long-range electrostatic repulsion induced by the positive charge on the protein surface and the existence of acetic acid solvation layer. Because -COOH in acetic acid releases free hydrogen ions more easily than -OH in ethanol, zein in acetic acid is more prone to protonation.

## Fabrication of zein-based nanoparticles and its challenges

The construction of zein particles based on unique solubility behavior of zein can be traced back to the 1990s. Zein particles were used as coating materials first in pharmaceutical industry, and then in drug delivery system (26). The preparation methods of zein particles have become rich and various, and can be broadly summarized as chemical crosslinking, antisolvent precipitation, supercritical antisolvent, antisolvent dialysis, pH driving, spray drying, and electrohydrodynamic atomization. Advantages and disadvantages of these methods have been summarized in Table 1.

**Table 1:** Advantages and disadvantages of the methods for preparing zein nanoparticles.

**Methods**	**Advantages**	**Disadvantages**
Chemical crosslinking	Load with hydrophobic actives	Produce micro-scale particles Certain safety hazard caused by introduction of toxic crosslinking agent
Emulsification/solvent evaporation	Encapsulate hydrophobic, hydrophilic and amphiphilic actives	Produce micro-scale particles
Antisolvent precipitation	Low cost Easy to operate	Utilization of numerous alcohols Discontinuous and unscalable process
Supercritical antisolvent	Easy to operate Continuous and large-scale production Produce particles with controllable size and shape Friendly to temperature-sensitive actives	Toxic solvent residues High cost, specific equipment required
Antisolvent dialysis	Low cost Optimization of antisolvent precipitation	Time-consuming
pH-driven	Low cost Easy to operateAvoid of organic solvents	Susceptibility of encapsulated actives to strong alkaline
Electrohydrodynamic atomization	Reduce preparation time Production of uniform particles	Specific equipment required

### Chemical crosslinking

**Figure 3:**
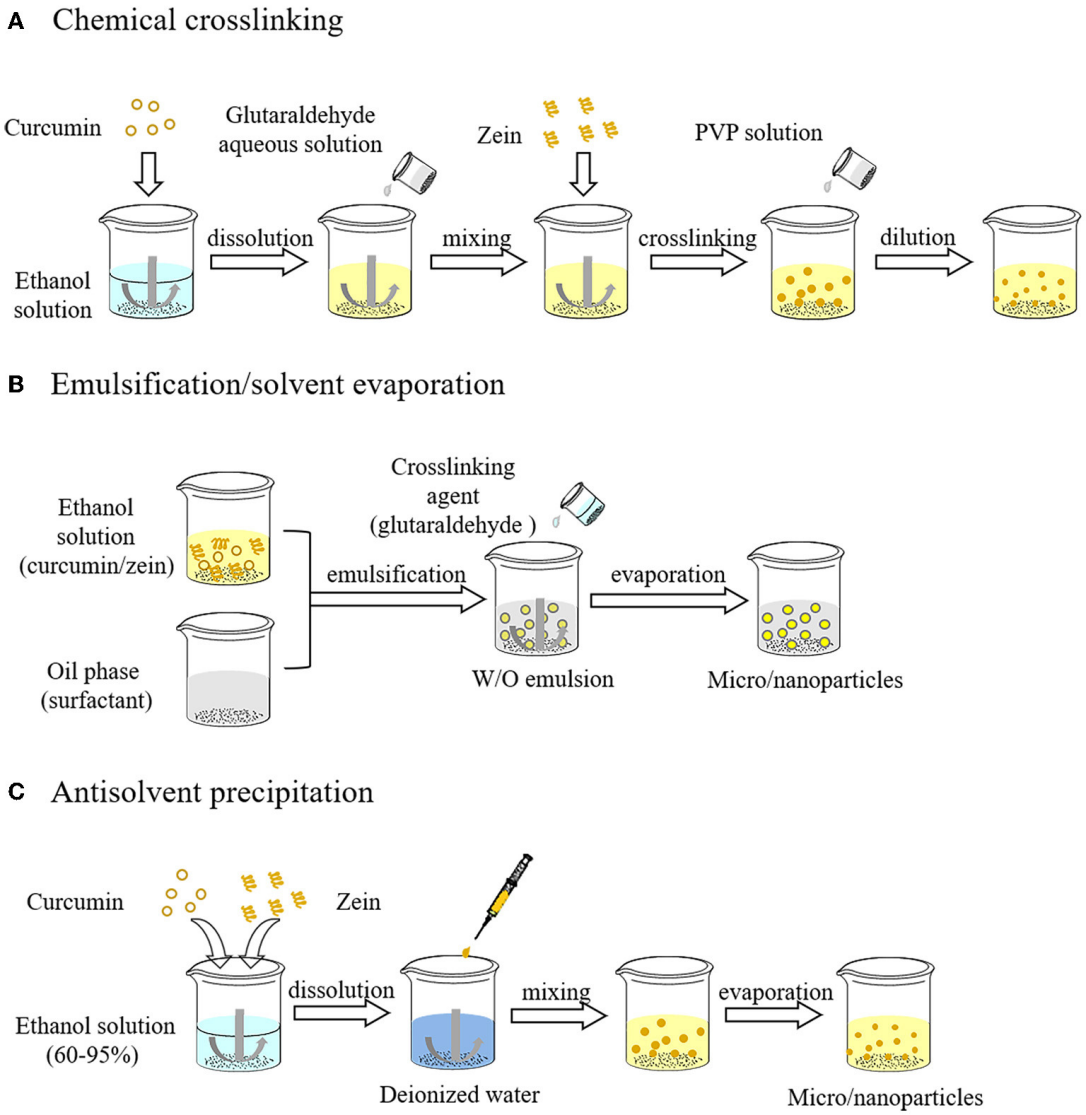
Preparation process of zein particles by chemical crosslinking **(A)**, emulsification/solvent evaporation **(B)** and antisolvent precipitation **(C)**.

Glutaraldehyde mediated crosslinking reaction between zein and other molecules (e.g., proteins, polysaccharides, polyphenols) or zein molecules themselves is one of the early methods for zein particles formation. This method originated in the 1980s (27, 28). Glutaraldehyde is a colorless and transparent oily liquid with a pungent odor. It is a commonly homo-bifunctional crosslinking agent that combines with phenol, imidazole, hydroxyl and amino through the aldehyde groups at both ends of its molecule. The crosslinking reaction between molecules endows zein a stronger aggregation trend when exposed to water solutions owing to the distinct surface hydrophobicity of zein, and increases its molecular weight, which promotes the self-assembly of zein into particles. However, zein particles formed by chemical crosslinking are usually micro-scale. The general process for the formation of zein particles loaded with hydrophobic active ingredients (e.g., curcumin) by chemical crosslinking was shown in Figure 3A. Curcumin as a model bioactive ingredient is firstly dissolved in an ethanol aqueous solution, then mixed with a small amount of a glutaraldehyde aqueous solution, and finally zein is added to cause a cross-linking reaction, resulting in the formation of internally encapsulated curcumin particles. Noticeably, the water volume accompanying glutaraldehyde aqueous solution added must be strictly controlled because the presence of a large amount of water will lead to the aggregation and precipitation of zein, thereby inhibiting the crosslinking efficiency. After the reaction, it was highly diluted with polyvinylpyrrolidone (PVP) solution and centrifugation, followed by ultrasound and freeze-drying to obtain zein particles.

There are some defects in the chemical crosslinking method. First, crosslinking reaction may produce adverse effects on the activity of ingredients encapsulated inside zein particles. Secondly, chemical crosslinking involves toxic agents, such as glutaraldehyde, which has a strong irritant effect on eyes, skin and mucous membrane, causing certain safety risks for the production process and the final products. Therefore, it is imperative to develop alternative green, safe and environmental-friendly chemical crosslinking agents.

### Emulsification/solvent evaporation

With emulsification/solvent evaporation, the zein-ethanol aqueous solution and the oil phase containing miscible surfactants are emulsified to form the water-in-oil emulsion as a template for carrier preparation. The internal aqueous phase of the emulsion can be solidified into rigid particles by solvent evaporation. Crosslinking agents (e.g., glutaraldehyde) are added before solvent evaporation to obtain crosslinked particles, which can moderately reduce the size of particles. The operation process of emulsification/solvent evaporation is shown in Figure 3B. Briefly, curcumin as a model active ingredient and zein are co-dissolved in an ethanol aqueous solution, emulsified with oil phase, and then a crosslinking agent is added into the emulsion. With the evaporation of ethanol, zein and curcumin inside emulsion droplets are subsequently solidified into rigid crosslinked particles (29). Finally, zein particles loaded with curcumin are obtained by filtration and removal of residual oil using suitable solvents. Emulsification/solvent evaporation method has been used to prepare zein particles loaded with aceclofenac, and their particle size ranges from 30 to 300 μm, which is suitable for oral administration.

### Antisolvent methods

#### Antisolvent precipitation method

Antisolvent precipitation, also known as liquid-liquid dispersion or phase separation, has been widely used for the preparation of zein nanoparticles by changing the polarity of solvents surrounding zein. As shown in Figure 3C, zein and curcumin are firstly dissolved in an ethanol aqueous solution (60%–95%, high solubility), which is then poured into or added dropwise into numerous water (as antisolvent) under magnetic stirring, so that the ethanol concentration decreased rapidly, resulting in supersaturation and precipitation of zein. Combined with the self-assembly characteristics, zein molecules aggregate into particles, and most of the curcumin spontaneously enters the hydrophobic core of zein particles. Subsequently, the ethanol is removed by rotary evaporation. Solvent polarity seriously affects the properties of zein particles. Ethanol, methanol and isopropanol can be used to prepare zein nanoparticles. The size of zein particles prepared from 80% alcohol solutions ranks by the alcohol type as ethanol>isopropanol>methanol, which may be due to the difference of solvent evaporation rate and zein solubility (30). In addition to the alcohol type, the initial concentration of alcohol and zein, the shear rate, the ratio of alcohol to water, pH, mixing mode and other added substances all can affect the particle properties.

#### Supercritical antisolvent method

In addition to using water as an antisolvent for the preparation of zein nanoparticles, supercritical fluid CO_2_ is also possible, due to its immiscibility with most polymers. The simple processing procedure of supercritical antisolvent method is that the co-solvent in atomized droplets is extracted by supercritical CO_2_ after the feedstock is continuously injected into supercritical CO_2_. Since most of the polymers are insoluble in CO_2_, the solubility of polymers decreases gradually, thus forming nuclei and gradually growing into nanoparticles or microparticles (31). The technology requires that the polymer be dissolved in one or more solvents (co-solvent), and these solvents can be miscible with supercritical CO_2_. Advantage of supercritical antisolvent method is that the critical temperature (31.1°C) and pressure (7.38 MPa) of CO_2_ can be easily achieved, and it is friendly to thermo-sensitive bioactives, so that they would not be degraded in the process of particle formation. As reported, supercritical CO_2_ has been used to form a micro/nano-particle delivery system for foods (32), so as to improve the physicochemical properties and health effects of active ingredients. Zein particles formed by supercritical CO_2_ antisolvent method were used to encapsulate lysozyme, which improved the antibacterial activity of lysozyme (33). Lutein/zein nanoparticles prepared by supercritical CO_2_ realized the controlled release of lutein (34).

Zein concentration, CO_2_ flow rate, alcohol type and concentration all affect the formation of zein particles. It is generally believed that large size particles are easily formed at high polymer concentrations due to the high viscosity of the solution, which further complicates the solution atomization. Meanwhile, the growth rate of particles is accelerated at high polymer concentrations. The increase of CO_2_ flow rate can promote the particle formation. In addition, the polarity of solvent affects the extraction efficiency of supercritical CO_2_, thus affecting the formation of zein particles. As for higher-polarity alcohols (methanol>ethanol>isopropanol), the extraction efficiency of supercritical CO_2_ (non-polar solvent) is lower, and the solidification rate of zein is slower. For example, zein dissolved in 90% methanol aqueous solution did not form particles by supercritical antisolvent technology, while zein dissolved in 90% isopropanol aqueous solution produced smaller particles than that dissolved in 90% ethanol aqueous solution. Reducing the water content of zein co-solvent is also helpful to supercritical CO_2_ extraction (mass transfer process) and speeds up the solidification of zein, thereby forming smaller and more uniform particles (31). It can be explained as follows: as the polarity of co-solvent increases (water or more polar alcohols are added), the mass transfer rate slows down due to the poor compatibility between CO_2_ and co-solvent. Particles are formed when the polymer precipitation kinetics can overcome the mass transfer limitation. The particle size of zein can be adjusted from micron to nanometer by changing the type and concentration of alcohol.

Supercritical antisolvent technology with CO_2_ has the advantages of mild operating temperature, friendly environment, simple operation and easy control. However, it is difficult to produce uniform zein nanoparticles unless 100% methanol is used as solvent and supercritical CO_2_ is loaded at a high flow rate. In this case, it will increase production costs and may cause toxicity due to residual organic solvents.

#### Antisolvent dialysis method

**Figure 4:**
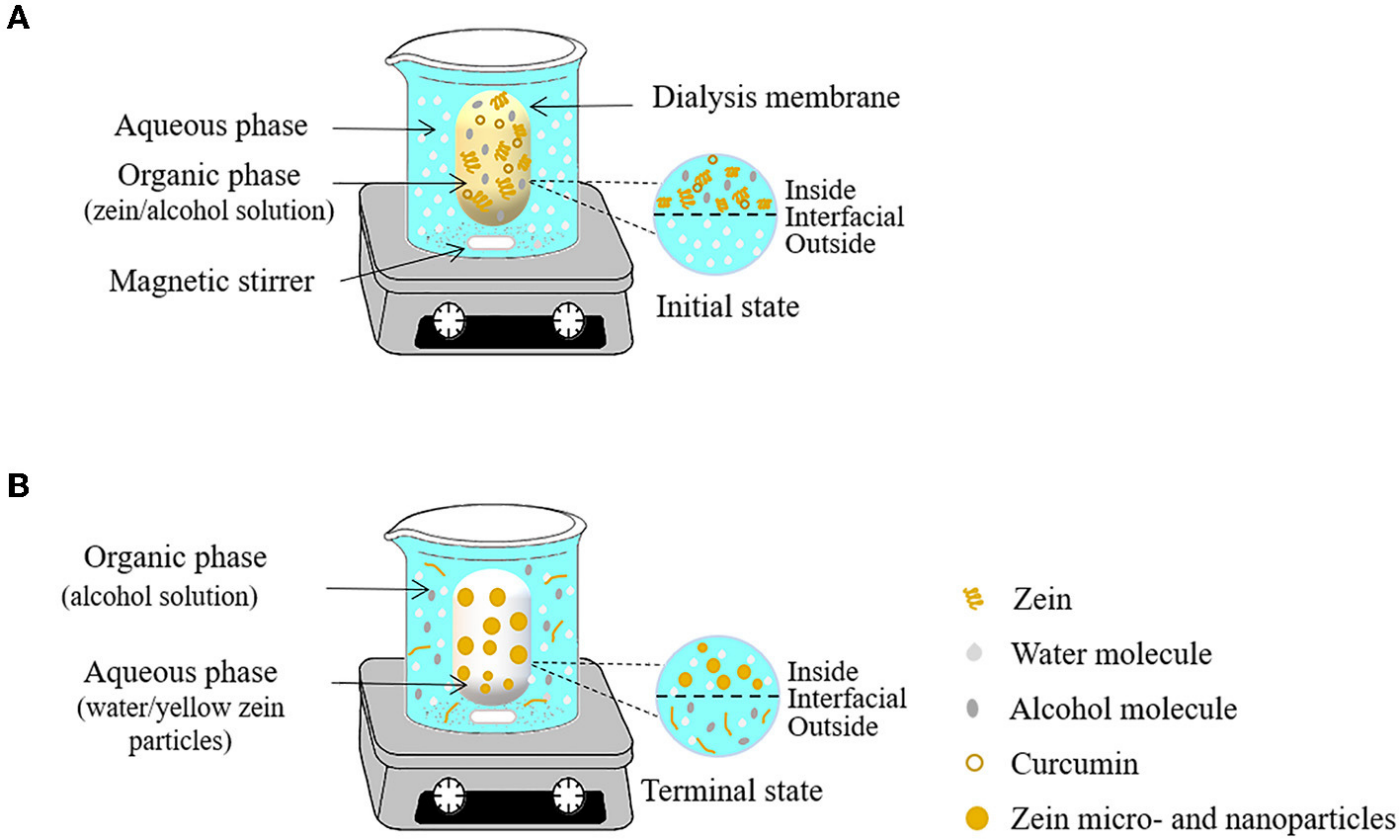
Schematic representation of antisolvent dialysis method for the obtaining of zein micro- and nanoparticles. At time 0 h **(A)** and after 12 h of the process **(B)**.

Antisolvent dialysis is a variant of antisolvent precipitation. Firstly, the biopolymer is miscible with the first solvent (organic phase) to form mother liquid. Then the first solvent is replaced by another solvent (aqueous phase) which is immiscible with the polymer to form a colloidal dispersion (35). The preparation of zein particles by antisolvent dialysis is mainly based on changes in polymer concentration gradient and solvent polarity. As shown in Figure 4, the dialysis bag containing an alcohol solution dissolving zein and curcumin is firstly placed in the aqueous phase, and then the organic solvent gradually diffused into the external aqueous phase through the semipermeable membrane. With this process, the change of the polar environment of zein induces the formation of zein particles. Compared with the antisolvent precipitation method, the advantage of the antisolvent dialysis method is that it only needs one-step to realize the formation of particles and solvent replacement simultaneously. Unlike antisolvent precipitation, additional operations (rotary evaporation) are required to remove organic solvents after particle formation.

### pH-driven method

Although the preparation of zein nanoparticles by antisolvent methods is simple and efficient, the method involves a large number of organic solvents, which would bring certain safety risks (flammability) and increase the cost due to the removal of alcohols after the particle formation. To solve these problems, Pan and Zhong innovatively prepared zein nanoparticles based on the solubility of zein in alkaline environment (pH ≥ 11.5) and insolubility in neutral aqueous solution, namely pH driven method, also known as pH cycling or pH transition method (36). Briefly, zein is dissolved in deionized water at pH 7.0, then the pH is adjusted to 12 with NaOH, and then adjusted back to pH 7.0 by HCl. During the pH changing from alkalinity to neutrality, zein is protonated and its solubility decreases gradually and aggregates to form particles. In this process, hydrophobic ingredients are encapsulated inside the particles. In the past few years, the pH-driven method has been widely used to prepare zein composite nanoparticles, including zein-rhamnolipid (37), zein-NaCas (38) and zein-WPI (39) composite nanoparticles. Compounding with other hydrophilic or charged food-grade biopolymers can adjust the aggregation behavior of single zein nanoparticles, thereby improving the stability of zein nanoparticles.

### Electrohydrodynamic atomization (EHDA)

EHDA, also known as electrospraying, has been used to prepare inorganic nanoparticles, drug nanoparticles and polymer drug delivery nanoparticles. The technique relies on the separation of liquids into tiny charged droplets under an electric field. The advantage of this technology is that it can produce spherical particles or fibrils with uniform morphology. The morphology and size of particles are seriously affected by processing parameters (e.g., polymer concentration, flow rate, voltage) (40). With the change of processing parameters, the particle size can change from hundreds of microns to several nanometers. The particle size is gradually enlarged with the increase of zein concentration (2.5–15%) (40).

Among the above methods, antisolvent precipitation and supercritical antisolvent method have attracted the attention of researchers owing to their simple operation and large-scale production potential.

## Zein-based nanoparticles as delivery system for hydrophobic bioactives

A variety of bioactive substances derived from plants, animals and microorganisms, such as the polyphenols (curcumin, resveratrol, and quercetin), and carotenoids (lycopene, lutein, astaxanthin), and vitamins A, D, E, K, have the wide range of health effects such as antioxidant, anti-inflammatory, anti-cancer and chronic disease prevention. However, these bioactive substances are susceptible to oxidation, crosslinking, or degradation in food processing, storage and digestion, and their absorption and utilization are limited because they are mostly insoluble in water. In order to improve the compatibility of these hydrophobic bioactives with food matrices and to ensure their stability during food processing and high bioavailability after digestion, it is necessary to add them to foods with the help of nano-sized delivery systems with good biocompatibility. Zein is an extremely promising carrier material for delivering hydrophobic bioactivity due to its excellent self-assembly characteristic, which makes it easy to form nanoparticles.

### General properties of zein-based nanoparticles

As an amphiphilic prolamin, zein with distinct hydrophobic and hydrophilic domains can self-assemble into nanoparticles, which have been extensively exploited in encapsulation and delivery applications for hydrophobic bioactives. Here, curcumin is used as a model hydrophobic active ingredient to discuss the effect of different zein-based nanoparticles on physicochemical and functional properties of hydrophobic bioactives. Zein nanoparticles (ZNPs) can effectively improve the stability of bioactives and facilitate their targeted delivery. Through antisolvent precipitation, the stability of curcumin encapsulated in ZNPs was improved at all physiologically relevant pH and UV irradiation (30). Moreover, the system has good mucoadhesion property *in vitro*, and the retention rate of curcumin was more than 60% when it was retained in the gastrointestinal tract for 150 min. The size of curcumin-loaded ZNPs prepared by electrofluid-driven atomization was within 175 to 900 nm, and increased with the increase of zein concentration. After 3 months of storage (23 °C, 43% relative humidity in the dark), no significant changes in the morphology of the nanoparticles or curcumin content were found. These nanoparticles showed good dispersion and coloring capacity in semi-skimmed milk compared to commercial curcumin (40). Furthermore, curcumin-loaded zein nanospheres formulated by antisolvent precipitation method improved liver targeting efficiency and antifibrotic activity of curcumin in a mouse model of carbon tetrachloride-induced liver fibrosis, compared with free curcumin (41).

### Zein peptides nanoparticles

Due to the high surface hydrophobicity of zein and its isoelectric point of 6.2, ZNPs without any stabilizer tend to aggregate at neutral pH (42). Therefore, their dispersion stability should be optimized for the delivery application of ZNPs. Proline-rich zein contains a large number of amphiphilic peptides (43). Amphiphilic zein peptide is also a potential delivery system due to its small size and self-assembly property in water solution (43–45). Under chemical or enzymatic treatments, glutamine and asparagine residues of zein undergo a deamidation reaction, which can increase the hydrophilicity of zein (46). Enzymatic method is desirable to modify zein structure. Transglutaminase (TGase), protease, peptidoglutaminase (PGase), glutaminase, and protein glutaminase (PG) can be used for protein deamidation (46). Alcalase-degraded zein peptides tend to self-assemble into micelles, leading to strong hydrophobic complexation with curcumin (44). Without any stabilizer, curcumin-loaded zein peptide nanoparticles exhibited a monodisperse size distribution (<50 nm) and enhanced water solubility (above 8,200-fold vs. free curcumin in water). In addition, this system showed improved storage stability, and more than 60% of curcumin was retained after 72 h of storage at ambient conditions (46). The deamidated zein peptide, produced by alkaline hydrolysis for 36 h, was dissolved together with curcumin in an alkaline aqueous solution and then acidified to induce the co-assembly of the peptide and curcumin to form complex nanoparticles (47). The nanoparticles displayed a curcumin loading capacity of 31.9%, *in vitro* bioaccessibility of 75%, and a significant antioxidant effect after oral administration in mice.

### Zein-based binary composite nanoparticles

The introduction of a biopolymer into the preparation process of ZNPs has been a prominent way to improve the stability and endue new functional properties. The resultant zein composite nanoparticles provide greater encapsulation efficiency (EE), better protection of the encapsulated bioactives from degradation and better controlled release during digestion than ZNPs alone. Zein-based binary composite nanoparticles fabricated with sodium caseinate (48, 49), WPI (6), chitosan (50, 51) and its derivative (52), pectin (53, 54), hyaluronan (55), gum Arabic (56), and carrageenan (57) have been constructed to improve their physicochemical and functional properties.

The addition of amphiphilic protein can reduce the surface hydrophobicity of ZNPs, and enhance their electrostatic and steric repulsion. Zein-caseinate composite nanoparticles were fabricated by a low-energy liquid-liquid dispersion method (48). It was found that the solubility, thermal and UV irradiation stability of encapsulated curcumin were improved, which may be ascribed to the electrostatic and steric repulsion between the zein particles provided by anionic caseinate. Moreover, the cellular studies showed that zein-caseinate nanoparticles with the same level of curcumin exhibited enhanced bioavailability, compared with free curcumin. In another study, WPI-zein composite nanoparticles were fabricated using the pH-driven method (6). Compared with individual WPI nanoparticles or ZNPs, the WPI-zein composite nanoparticles showed improved physicochemical properties. ZNPs aggregated at pH 7.0 and WPI nanoparticles showed relatively poor resistance against high temperature (80 °C).

Many researches also focus on the effects of polysaccharide on the formation, properties and stability of ZNPs. For instance, water-soluble chitosan derivatives (N-(2-hydroxyl)propyl-3-trimethyl ammonium chitosan chloride, HTCC) with different molecular weight were synthesized and used to prepare zein-HTCC nanoparticles (52). HTCC coating was helpful to obtain high EE of curcumin, and EE was positively correlated with the molecular weight of HTCC due to the longer chain of the HTCC molecule could entrap more curcumin. In addition, zein composite nanoparticles with a unique structure were fabricated with chitosan in step-by-step to enhance the loading space of bioactives (51). Curcumin-zein-resveratrol-chitosan composite nanoparticles appeared as the three-dimensional network structure, which was proved to be an effective carrier for greatly improving the physical-, light-, thermal-, and storage stability of both curcumin and resveratrol. Shellac, a resin secreted by the female lac beetle, is insoluble in water but highly soluble in anhydrous ethanol. Zein-shellac binary nanoparticles provided a stronger encapsulation ability of curcumin. After combining shellac, the EE of curcumin increased from 82.7% to 93.2%. Moreover, zein-shellac nanoparticles exhibited improved photochemical, thermal stability and strong curcumin sustained-release ability (in both PBS and simulated gastrointestinal tract), which were ascribed to compact structure induced by binary complexation between zein and shellac (58).

Furthermore, some surfactants can stabilize ZNPs against aggregation by reducing hydrophobic attraction and increasing electrostatic and steric repulsion. Dai et al. (37) successfully prepared zein-rhamnolipid composite nanoparticles using a pH-driven method, which exhibited good stability from pH 3 to 8 and at low salt concentrations (0–100 mM NaCl, pH 7). Nevertheless, they were unstable under extremely acidic conditions or at high ionic strengths due to the suppression of these electrostatic forces. Interestingly, ZNPs stabilized by different levels of surfactants may have different structures, which affect EE of curcumin and related physicochemical properties of nanoparticles. At a relatively low level of lecithin, the nanoparticles tended to aggregate due to embedded alkyl chains of lecithin in the hydrophobic region of zein. With the further increase in lecithin concentration, the strong hydrophobic interaction between the alkyl chain of lecithin with zein and curcumin led to compact structure and smaller size of zein-lecithin composite nanoparticles (59). There are two distinct binding behaviors of curcumin in ZNPs depending on the surfactant concentration (60). At Tween 20 concentration below 0.2 g/L, curcumin was bound in the hydrophobic cavities of the ZNPs; at Tween 20 concentration above 0.2 g/L, some curcumin was also bound in the micellar-like Tween 20 aggregates of the particles.

Cross-linking is another option that may improve the stability and delivery potentials of ZNPs. It is known that zein has some resistance to acid environment and pepsin digestion, but is susceptible to pancreatic enzymes. In terms of the stability under simulated gastric and intestinal conditions, tannic acid cross-linked ZNPs were more resistant against digestion by pancreatin (which was able to degrade α-zein dimers) than non-crosslinked ones (61).

### Zein-based ternary composite nanoparticles

Although zein-based binary composite nanoparticles show desirable stability, many reports indicate that the development of ternary composite nanoparticles may be a more promising means of encapsulating, protecting and delivering bioactives. Zein/polysaccharide/surfactant ternary composite nanoparticles were prepared by antisolvent co-precipitation recently. The encapsulation efficiency of curcumin in zein-propylene glycol alginate-rhamnolipid (or lecithin) ternary composite nanoparticles was higher than that in pure zein or binary zein-propylene glycol alginate nanoparticles. Moreover, the presence of the surfactants significantly improved bioaccessibility of curcumin (62). In another study, ternary composite nanoparticles consisting of zein, chondroitin sulfate (CS) and sophorolipid enhanced EE, chemical stability, aqueous solubility and anticancer activity *in vitro* against HepG2, MCF-7 and HeLa cells of curcumin, compared with ZNPs and zein-CS nanoparticles (63), which were attributed to amphiphilic Spl. Its hydrophilic disaccharide sophorose could produce hydrogen bonding with –OH of CS, and its lipophilic long monounsaturated fatty acid chain could absorb on the hydrophobic region of zein by hydrophobic interaction. Zein/polysaccharide/protein ternary composite nanoparticles have also been extensively explored. The electrostatic biopolymer coatings consisting of a mixture of sodium alginate (70%) and fish gelatin (30%) improved the pH, salt, and thermal stability of ZNPs, and produced a high bioaccessibility of curcumin *in vitro* and antioxidant capacity (64). The re-dispersibility of the freeze-dried nanoparticles was enhanced due to hydrophilic sodium caseinate and sodium alginate adsorbed sufficiently on the surface of the ZNPs, resulting in a decrease in zein surface hydrophobicity (65).

In the ternary composite nanoparticles, the type of polysaccharide critically influences the stability of the system. Partial replacement of the pectin (low charge density) with alginate (high charge density) to form the shell in the ternary composite nanoparticles improved aggregation stability at pH 5 to 7 and at high ionic strengths (2,000 mM NaCl), and the encapsulated curcumin exhibited better antioxidant activity (66). In another study, ternary composite nanoparticles composed of zein, hyaluronic acid and chitosan were prepared using the layer-by-layer technique to co-deliver curcumin and piperine, the nanoparticles were able to control the release of the bioactive ingredients in simulated gastrointestinal conditions and protected them from chemical degradation when exposed to UV light, high temperature, or long-term storage by manipulating the number of layers (67). Chitosan with lower molecular weight is a suitable outer coating to protect and control the release rate of both bioactive ingredients during simulated digestion than either medium- or high-molecular weight chitosan. In addition, crosslinking can significantly influence the release sustainability of active ingredients by modifying carrier microstructures (19). Caseinate-zein-pectin composite nanoparticles were prepared *via* a pH- and heating-induced electrostatic deposition, and 1-ethyl-3-(3-dimethyl aminopropyl) carbodiimide and N-hydroxysuccinimide (EDC/NHS) as chemical cross-linkers were used to covalently bridge the protein and polysaccharide layers. The obtained nanoparticles showed improved stability under simulated gastrointestinal conditions (68). Oxidized dextran as a macromolecular crosslinker and coating can also successfully apply to stabilize the zein-caseinate composite nanoparticles, which not only enhanced the colloidal stability of composite nanoparticles but also improved the controlled release rate of curcumin under simulated gastrointestinal conditions (69). We summarize the physicochemical properties of different zein nanoparticles loaded with hydrophobic bioactives consisting of zein with proteins, polysaccharides, polyphenols, and surfactants reported in recent years (Supplementary Table S1, Supporting Information).

## Other Zein-based nanocarriers as delivery systems for hydrophobic bioactives

**Figure 5:**
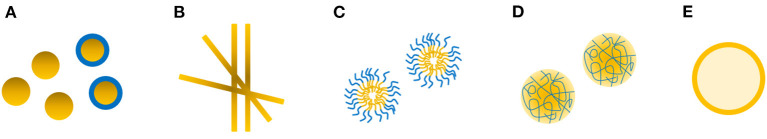
Schematic presentation of common zein-based nanosized delivery systems including nanoparticles **(A)**, nanofibers **(B)**, nanomicelles **(C)**, nanogels **(D)**, nanoemulsions **(E)**.

In addition to zein-based nanoparticles detailed above, nanofibers, nanomicelles, nanogels based on zein are excellent candidates as delivery systems for bioactives (Figure 5).

### Zein-based nanofibers

Electrospinning is a unique and cost-effective route to prepare nanofibers, and large surface area-to-volume ratio nanofibers can be provided by adjusting the preparation parameters (electrostatic field strength, flow rate, solution concentration, etc.,). Ultrafine zein fluorescence nanofibers loaded with curcumin were developed by electrospinning (70). It was found the diameter of zein nanofibers increased slightly from 250 nm to 300 nm with the increase of the concentration of embedded curcumin (0–10%), the morphology and distribution of nanofibers did not change significantly, and curcumin aggregates were also not found in fibers surfaces, indicating curcumin was well-embedded within the fibers. It was also confirmed in the study of Deng et al. (71) that the embedded bioactive ingredient did not affect the appearance and morphology of nanofibers. However, pure zein nanofibers have poor mechanical properties and solvent resistance (72). Hybrid electrospinning of zein and other biopolymers has been considered as an effective mean to remedy these deficiencies. It has been reported that hybrid electrospinning of zein and gelatin enhanced the mechanical properties of pure zein nanofibers, elongation at break and elastic modulus were up to 87%, 72.1 MPa respectively (73). Additionally, both the glucose cross-linked gelatin/zein nanofibers and zein/tragacanth nanofibers with core-shell structure showed good sustained release properties (71, 74). Therefore, zein-based nanofibers prepared by electrospinning technology could be a potential candidate for delivering hydrophobic bioactives.

In addition, curcumin-loaded zein nanofibers exhibited excellent antibacterial activity, and the inhibition efficiency against food-borne pathogens increased with the increase of curcumin content (75–78). In addition, curcumin-loaded nanofibril films prepared by dispersing electrospun zein into konjac and glucomannan solution exhibited better hydrophobicity, water resistance property and excellent antibacterial activity, which can be used as food packaging materials (38).

### Zein-based nanomicelles

Polymer micelles are also important nano-sized delivery systems, formed through spontaneous self-assembly of amphiphilic block copolymers into specific supramolecularly ordered aggregates for improving stability and bioavailability of active ingredients (79). In aqueous solution, polymer self-assembles into a core-shell architecture, in which the hydrophobic segment forms the core and the hydrophilic segment forms the external corona, when the copolymer concentration approaches the critical micelle concentration (80). The hydrophobic active ingredients can be located in the micelle core regardless of inclusion or bonding, and the hydrophilic shell of the micelles increases the water solubility of the hydrophobic active ingredients. Many amphiphilic materials have been used to synthesize micelles, including synthetic polymers (81–83) and natural polymers (84–86).

Naturally accessible proteins and polysaccharides are attractive alternatives to synthetic polymers due to their good biocompatibility and low cost. Among them, zein with inherent hydrophobic regions have been widely discussed. In a previous study, methoxy polyethylene glycol grafted α-zein micelles (mPEG-g-α-zein) exhibited a sustained release effect of curcumin and low cytotoxicity (87). Although some PEG-modified products have been approved by FDA and other regulatory agencies and successfully used in the clinic, some researchers are still worried about whether it will cause subsequent toxicity and other side effects after long-term accumulation in the body (88). For this reason, zein-lactoferrin micelles were prepared *via* carbodiimide coupling reaction between the primary amine of zein and the carboxyl group of lactoferrin, which greatly enhanced the tumor targeting of the loaded drug (89). The effect could be attributed to: (i) the hydrophobic zein core in micelles had high drug loading; (ii) the hydrophilic lactoferrin shell enhanced the stability of micelles; (iii) lactoferrin provided the tumor targeting (90, 91). Similarly, the hydrophilic polysaccharide chondroitin sulfate (ChS) with the CD44-mediated tumor targeting effect was also used to prepared zein-ChS micelles (92). In addition, superhydrophilic zwitterionic polymer, poly(sulfobetaine methacrylate) (PSBMA) was also used to synthesize zein-PSBMA micelles. The micelles significantly improved the water solubility of curcumin, and curcumin loaded in micelles exhibited better stability, cellular uptake, cytotoxicity against cancer cells compared with native curcumin (93). Therefore, the amphiphilic zein-based micelles have great potential for delivering bioactives due to their outstanding stability and targeting capability.

### Others

Nanogel is a nanosized hydrogel, and it possess the features of hydrogels and nanoparticles, which is an excellent active ingredient delivery system with high loading and controlled release performance (94, 95). Zein-hyaluronic acid nanogels loaded with curcumin exhibited high anticancer activity against the CT26 colorectal cancer cells due to the enhanced targetability to CD44 receptor by the presence of hyaluronic acid (96). On the other hand, all these nano-sized zein materials could be used as emulsifiers to construct stable nanoemulsions for delivering curcumin, either oil-in-water single or multi-layer nanoemulsions (70, 97, 98).

## Zein-based hybrid nano-delivery systems

Nano-sized delivery systems such as nanoparticles, nanofibers, nanomicelles, nanogels and nanoemulsions have been proved to possess several protective functional advantages for bioactives. Comparatively, zein-based nanoparticles have higher barrier properties than oil-in-water nanoemulsion in limiting peroxyl radical-induced oxidation processes of encapsulated curcumin (99). However, these two encapsulation systems were ineffectiveness in limiting oxygen permeation. In a comparative study of improving curcumin bioavailability by three potential delivery systems (ZNPs, nanoemulsions, and phospholipid nanoparticles), ZNPs were able to incorporate the highest level of curcumin per unit mass of particles. Nevertheless, nanoemulsions appeared to be the most effective at promoting both the chemical stability and solubilization of curcumin at an equal initial curcumin level, under simulated gastrointestinal conditions (100). Chuacharoen and Sabliov (101) also synthesized nanoemulsions and nanoparticles with entrapped curcumin at the same level of surfactant concentration. Compared with nanoemulsions, zein-based nanoparticles were found to be the better carriers based on their storage stability and sustained release profile.

**Figure 6:**
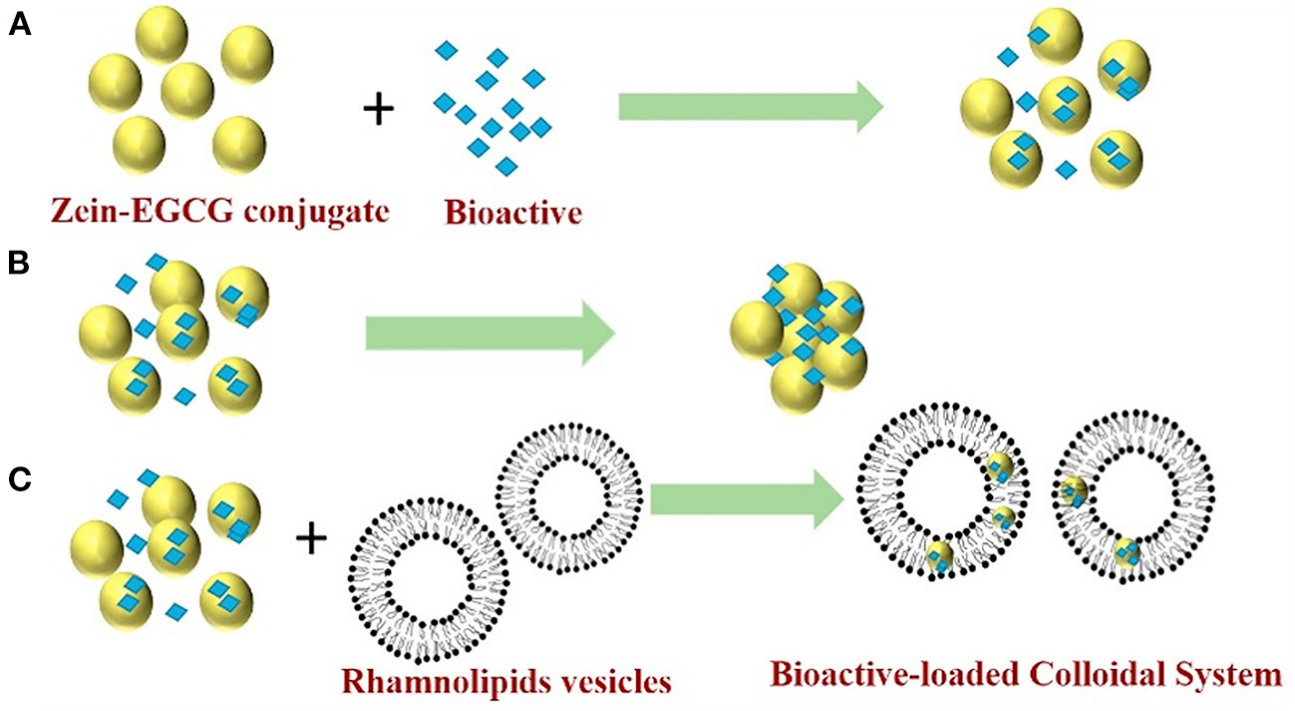
Proposed mechanisms for a mixed colloidal delivery system consisting of zein-EGCG conjugate nanoparticles and rhamnolipids vesicles. Zein-EGCG conjugate interacts with bioactive and self-assembles to form nanoparticles that embedded bioactive inside by antisolvent **(A)**; In the absence of surfactant, the nanoparticles aggregate due to hydrophobic interactions of zein in water solution **(B)**; In the presence of rhamnolipids vesicles, the nanoparticles do not aggregate because the coating of surfactant reduces hydrophobic interactions and increases the electrostatic and steric effects between nanoparticles **(C)**. Reprinted from (102) with permission from Elsevier.

To fully utilize the advantages of different systems, is it possible to achieve better encapsulation and delivery of hydrophobic bioactives if two or more systems are combined? The advantages and disadvantages of different nano-sized delivery systems are summarized in Table 2. Reportedly, mixing curcumin-loaded ZNPs with lipid nanoparticles can increase the bioaccessibility and chemical stability of lipophilic bioactive agents (100). ZNPs can be designed to protect the curcumin from chemical degradation, whereas another type of nanoparticles (like lipid nanoparticles) can be designed to rapidly digest within the gastrointestinal tract and form mixed micelles that can solubilize and transport the hydrophobic curcumin. In a another study (102), zein-EGCG conjugate nanoparticles (Figure 6A) aggregated in water solution due to hydrophobic interaction of protein in the absence of surfactant (Figure 6B). However, mixing the nanoparticles with rhamnolipids vesicles formed uniform and stable bioactive-loaded colloids nanoparticles due to rhamnolipids adsorption and hydrophobic effect around the protein nanoparticles (Figure 6C). Furthermore, the mixed colloidal delivery system improved the digestibility of the nanoparticles and bioaccessibility of curcumin and resveratrol.

**Table 2:** Advantages and disadvantages of different nano-sized delivery systems.

**Systems**	**Advantages**	**Disadvantages**
Nanoparticles	Good resistance to oxidation High encapsulation efficiency Controlled release and targeting effect	Inability to limit oxygen permeation Complex modifications Some methods are not suitable for large-scale production
Nanofibers	High loading capacity and sustainable release Easy to large-scale production	Special equipment Strict processing parameters Solvent residue
Nanomicelles	Strong thermodynamic stability High loading capacity and sustainable release Long blood circulation time *in vivo* Able to avoid phagocytosis by immune system cells without additional modifications	Potential side effects from modifier involved Instability in blood dream
Nanogels	High loading capacity and responsive release Wide delivery route	Special equipment Potential side effects from the crosslinker involved
Nanoemulsions	Improved chemical stability and solubilization High bioavailability	Thermodynamically unstable Inability to limit oxygen permeation

## Conclusions and future trends for zein-based nano-sized carriers

Zein is considered as an ideal biopolymer for constructing nanoscale carriers owing to its intrinsic hydrophobic and self-assembly properties. Developing zein nanocarriers can provide controlled release possibilities and enhance the oral bioavailability of hydrophobic bioactives. The colloid stability of zein-based delivery system can be improved using chemical crosslinking, coating and complexation by introducing other biopolymers (e.g., proteins, polysaccharide, phenols, surfactant). Most of these modification strategies can further enhance the encapsulation and delivery efficiency of bioactives.

Nevertheless, some issues remain to be considered for future study: (i) The appropriate loading level of nutrient and the actual sensory properties of these carriers for commercial foods should be determined; (ii) Various factors introduced in the process of carrier construction should be carefully considered, such as the involvement of toxic organic solvents; (iii) Whether the effects of the observations would also occur under more realistic physiological gastrointestinal conditions should be determined *via* using animal or human feeding models. In the future, zein based delivery systems will be designed to be functional and even more complex based on specific needs. More studies will focus on elucidating the efficacy and toxicity of these carriers as well as the biological fate of oral administration under physiological conditions. The preparation process requires more work based on a comprehensive analysis not only of their reproducibility, biocompatibility and immunogenicity, but also their scalability, storage stability and cost.

## Author contributions

XY: writing—original draft preparation and revision. ML: writing—original draft preparation. XX and XL: project administration. FL: writing—review and editing, project administration, and funding acquisition. All authors contributed to the article and approved the submitted version.

## Funding

This research was supported by Innovation Talents Promotion Plan of Shaanxi Province [No. 2020KJXX-034], the Fundamental Research Funds for the Central Universities [No. 2452020008], and the Breeding Plan of Shandong Provincial Qingchuang Research Team (2021-Innovation Team of Functional Plant Protein-Based Food).

## Conflict of interest

The authors declare that the research was conducted in the absence of any commercial or financial relationships that could be construed as a potential conflict of interest.

## Publisher's note

All claims expressed in this article are solely those of the authors and do not necessarily represent those of their affiliated organizations, or those of the publisher, the editors and the reviewers. Any product that may be evaluated in this article, or claim that may be made by its manufacturer, is not guaranteed or endorsed by the publisher.
