# Supplementary material for: Zein-based nano-delivery systems for encapsulation and protection of hydrophobic bioactives: A review
Source: Front Nutr. 2022 Sep 28;9:999373. doi: 10.3389/fnut.2022.999373 (PMC9554640; doi:10.3389/fnut.2022.999373)
Supplement: Supplementary file 1 [file Table_1.docx]

**Table S1** Summary of literature on zein-based nanoparticles for hydrophobic bioactives.

| Bioactives | Modifier | Preparation method | Size | Encapsulation Efficiency | Highlights | Reference |
| --- | --- | --- | --- | --- | --- | --- |
| Curcumin | Caseinate | Antisolvent precipitation | 187.16 nm | 95.55% | Improved water dispersibility , thermal- and UV irradiation stability and bioavailability of curcumin. | (1) |
| Curcumin/EGCG | Caseinate | Antisolvent precipitation | 100-200 nm | 96.2% for curcumin | Improved water dispersibility and DPPH radical scavenging capacity of curcumin. | (2) |
| Curcumin | Whey protein isolate | pH-driven method | About 90 nm | 45.28%-84.62% | Improved water dispersibility and thermal stability of curcumin. | (3) |
| Curcumin | Chitosan | Antisolvent precipitation | 162.07 nm | 94.67% | Good controlled release properties of curcumin at pH 4.0 compared to pH 3.0 and 7.0. | (4) |
| Curcumin/ resveratrol | Chitosan | Antisolvent precipitation | 173.4 nm | 91.3% for curcumin/ 82.1% for resveratrol | The half-life time of curcumin and resveratrol were extended by 4.5- and 1.9-fold during photodegradation and the retention rates were increased by 3.7- and 1.1-fold during thermal degradation than free forms. | (5) |
| Curcumin | N-(2-hydroxyl)propyl-3-trimethyl ammonium chitosan chloride | Antisolvent precipitation | 66-177 nm | 94.9% | Improved stability of curcumin against heat and UV light. | (6) |
| Curcumin | Citrus pectin | Antisolvent precipitation | 250 nm | >86% | Good water dispersibility and encapsulation capacity of curcumin. | (7) |
| Curcumin | Pectin from *Akebia trifoliata var. australis* fruit peel | Antisolvent precipitation | 230 nm | 89.65% | Increased the solubility, stability, antioxidant activity, and *in vitro* bioavailability of the curcumin. | (8) |
| Curcumin | Hyaluronic acid | Antisolvent precipitation | 140.37 nm | 95.03% | Improved the stability of curcumin to heat and UV light and controlled its release in simulate gastrointestinal tract. | (9) |
| Curcumin and quercetagetin | Hyaluronic acid | Antisolvent precipitation | 231.2 nm | 69.8% for curcumin/ 90.3% for quercetagetin | Prevented both curcumin and quercetagetin from light and thermal degradation and controlled their release in simulated gastrointestinal tract. | (10) |
| Tocopherol | Gum Arabic | Antisolvent precipitation | 120-170 nm | >90% | Controlled release of tocopherol. | (11) |
| Curcumin | Gum Arabic | Antisolvent precipitation | 50-250 nm | - | Good stability in pH 5-8 and 0-8 mM NaCl environment. | (12) |
| Curcumin/ piperine | Carrageenan | Antisolvent precipitation and Ca^2+^crosslinking | 364.9 nm | - | Crosslinking of the carrageenan improved photo- and thermal-stability and delayed the release of curcumin. | (13) |
| Curcumin | Tea saponin | pH-driven method | 100-250 nm | 83.73% | The solubility and bioaccessibility of encapsulated curcumin increased by about 290-fold and 5-fold respectively, compared with free curcumin. | (14) |
| Quercetin | Soluble soybean polysaccharide | Antisolvent precipitation | About 200 nm | 82.5 % | Enhanced the photochemical stability of quercetin. | (15) |
| Lutein | Glucosamine | Transglutaminase-induced glycosylation | <200 nm | >80% | Improved the solubility and antioxidant activity of lutein. | (16) |
| Curcumin | Rhamnolipid | pH-driven method | About 100 nm | 81.8%-96.2% | Exhibited good stability across a range of pH 3-8, at low salt concentrations (0-100 mM NaCl, pH 7) and relatively low temperatures (37 and 55°C). | (17, 18) |
| Curcumin | Lecithin | Antisolvent precipitation | 130-424 nm | 99.83% | Improved the stability against thermal treatment, UV irradiation and high ionic strength. | (19) |
| Curcumin | Tween 20 | Antisolvent precipitation | 137 nm | 97.7% | Increased stability and radical scavenging ability of curcumin. | (20) |
| Curcumin | Tannic acid | Antisolvent precipitation and Na_2_CO_3_ as sacrificing template | ＜100 nm | 95.82% | Enhanced the stability of curcumin in simulated intestinal digestion. | (21) |
| Curcumin | Propylene glycol alginate and surfactant (rhamnolipid/lecithin) | Antisolvent precipitation | 200-500 nm | 92%-94% | The presence of the surfactants significantly improved the photo-stability and bioaccessibility of curcumin. | (22) |
| Curcumin | Caseinate/alginate | Antisolvent precipitation | 268 nm | 92% | Improved water solubility, photochemical stability, antioxidant activity and control release properties of curcumin. | (23) |
| Curcumin and piperine | Hyaluronic acid/chitosan | Antisolvent precipitation and layer-by-layer technique | 599 nm | 90.4% for curcumin and 86.4% for piperine | The layer-by-layer of coatings increased the physicochemical stability and controlled the release of the bioactives in simulated digestion. | (24) |
| Curcumin | Caseinate and polysaccharides (gum arabic/pectin/ carboxymethyl cellulose) | pH- and heating-induced electrostatic adsorption and EDC/NHS crosslinking | 147-247 nm | 80% | Compared to gum arabic, pectin and CMC were favorable to form crosslinked nanoparticles with higher EE and better physicochemical property. | (25) |
| Curcumin | Caseinate/oxidized dextran | pH- and heating-treatment | 150 nm | >90% | Improved the stability of nanoparticles and controlled release of curcumin in simulated gastrointestinal conditions. | (26) |
| Curcumin | Caseinate/pectin | Antisolvent precipitation and Na_2_CO_3_ as sacrificing template, pH- and heating induced electrostatic adsorption of pectin | 112 nm | 92% | Provided sustained release of curcumin in simulated gastrointestinal conditions. | (27) |
| Resveratrol | Alginate/chitosan | Antisolvent precipitation | 100-300 nm | >70% | Improved stability and bioaccessibility of resveratrol. | (28) |
| Quercetin | Chitosan/pectin | Antisolvent precipitation and Na_2_CO_3_ as sacrificial templet | <300 nm | 86-94% | Improved the physicochemical stability and antioxidant activity of quercetin. | (29) |

1. Xue J, Zhang Y, Huang G, Liu J, Slavin M, Yu L. Zein-caseinate composite nanoparticles for bioactive delivery using curcumin as a probe compound. *Food Hydrocoll* (2018) 83:25-35.
2. Yan X, Zhang X, McClements DJ, Zou L, Liu X, Liu F. Co-encapsulation of epigallocatechin gallate (EGCG) and curcumin by two proteins-based nanoparticles: Role of EGCG. *J Agri Food Chem* (2019) 67(48):13228-13236.
3. Zhan X, Dai L, Zhang L, Gao Y. Entrapment of curcumin in whey protein isolate and zein composite nanoparticles using pH-driven method. *Food Hydrocoll* (2020) 106:105839.
4. Li M, Chen L, Xu M, Zhang J, Wang Q, Zeng Q, Wei X, Yuan Y. The formation of zein-chitosan complex coacervated particles: Relationship to encapsulation and controlled release properties. *Int J Biol Macromol* (2018) 116:1232-1239.
5. Chen S, Han Y, Jian L, Liao W, Zhang Y, Gao Y. Fabrication, characterization, physicochemical stability of zein-chitosan nanocomplex for co-encapsulating curcumin and resveratrol. *Carbohyd* *Polym* (2020) 236:116090.
6. Liang H, Zhou B, He L, An Y, Lin L, Li Y, Liu S, Chen Y, Li B. Fabrication of zein/quaternized chitosan nanoparticles for the encapsulation and protection of curcumin. *RSC Adv* (2015) 5(18):13891-13900.
7. Hu K, Huang X, Gao Y, Huang X, Xiao H, McClements DJ. Core-shell biopolymer nanoparticle delivery systems: Synthesis and characterization of curcumin fortified zein-pectin nanoparticles. *Food Chem* (2015) 182:275-281.
8. Cai T, Xiao P, Yu N, Zhou Y, Mao J, Peng H, Deng S. A novel pectin from Akebia trifoliata var. australis fruit peel and its use as a wall-material to coat curcumin-loaded zein nanoparticle. *Int J Biol Macromol* (2020) 152:40-49.
9. Chen S, Han Y, Sun C, Dai L, Yang S, Wei Y, Mao L, Yuan F, Gao Y. Effect of molecular weight of hyaluronan on zein-based nanoparticles: Fabrication, structural characterization and delivery of curcumin. *Carbohyd Polym* (2018) 201(17):599-607.
10. Chen S, Han Y, Huang J, Dai L, Du J, McClements DJ, Mao L, Liu J, Gao Y. Fabrication and characterization of layer-by-layer composite nanoparticles based on zein and hyaluronic acid for codelivery of curcumin and quercetagetin. *ACS Appl Mater Inter* (2019) 11(18):16922-16933.
11. Li J, Xu X, Chen Z, Wang T, Wang L, Zhong Q. Biological macromolecule delivery system fabricated using zein and gum arabic to control the release rate of encapsulated tocopherol during in vitro digestion. *Food Res Int* (2018) 114:251-257.
12. Chen G, Fu Y, Niu F, Zhang H, Li X, Li X. Evaluation of the colloidal/chemical performance of core-shell nanoparticle formed by zein and gum Arabic. *Colloid Surface A* (2019) 560:130-135.
13. Chen S, Li Q, Julian D, Han Y, Dai L, Mao L, Gao Y. Co-delivery of curcumin and piperine in zein-carrageenan core-shell nanoparticles: Formation, structure, stability and in vitro gastrointestinal digestion. *Food Hydrocoll* (2020) 99:105334.
14. Yuan Y, Xiao J, Zhang P, Ma M, Wang D, Xu Y. Development of pH-driven zein/tea saponin composite nanoparticles for encapsulation and oral delivery of curcumin. *Food Chem* (2021) 364:130401.
15. Li H, Wang D, Liu C, Zhu J, Fan M, Sun X, Wang T, Xu Y, Cao Y. Fabrication of stable zein nanoparticles coated with soluble soybean polysaccharide for encapsulation of quercetin. *Food Hydrocoll* (2019) 87:342-351.
16. Chang Y, Jiao Y, Li D, Liu X, Han H. Glycosylated zein as a novel nanodelivery vehicle for lutein. *Food Chem* (2022) 376:131927.
17. Dai L, Zhou H, Wei Y, Gao Y, McClements DJ. Curcumin encapsulation in zein-rhamnolipid composite nanoparticles using a pH-driven method. Food Hydrocoll (2019) 93:342-350.
18. Dai L, Li R, Wei Y, Sun C, Mao L, Gao Y. Fabrication of zein and rhamnolipid complex nanoparticles to enhance the stability and in vitro release of curcumin. *Food Hydrocoll* (2018) 77:617-628.
19. Dai L, Sun C, Li R, Mao L, Liu F, Gao Y. Structural characterization, formation mechanism and stability of curcumin in zein-lecithin composite nanoparticles fabricated by antisolvent co-precipitation. *Food Chem* (2017) 237:1163-1171.
20. Wang X, Huang H, Chu X, Han Y, Li M, Li G, Liu X. Encapsulation and binding properties of curcumin in zein particles stabilized by Tween 20. *Colloid Surface A* (2019) 577:274-280.
21. Hu S, Wang T, Fernandez ML, Luo Y. Development of tannic acid cross-linked hollow zein nanoparticles as potential oral delivery vehicles for curcumin. *Food Hydrocoll* (2016) 61:821-831.
22. Dai L, Wei Y, Sun C, Mao L, McClements DJ, Gao Y. Development of protein-polysaccharide-surfactant ternary complex particles as delivery vehicles for curcumin. *Food Hydrocoll* (2018) 85(17):75-85.
23. Liu Q, Jing Y, Han C, Zhang H, Tian Y. Encapsulation of curcumin in zein/caseinate/sodium alginate nanoparticles with improved physicochemical and controlled release properties. Food Hydrocoll (2019) 93:432-442.
24. Chen S, McClements DJ, Jian L, Han Y, Dai L, Mao L, Gao Y. Core-shell biopolymer nanoparticles for co-delivery of curcumin and piperine: Sequential electrostatic deposition of hyaluronic acid and chitosan shells on the zein core. *ACS Appl Mater Inter* (2019) 11(41):38103-38115.
25. Chang C, Wang T, Hu Q, Luo Y. Caseinate-zein-polysaccharide complex nanoparticles as potential oral delivery vehicles for curcumin: Effect of polysaccharide type and chemical cross-linking. *Food Hydrocoll* (2017) 72:254-262.
26. Rodriguez NJ, Hu Q, Luo Y. Oxidized dextran as a macromolecular crosslinker stabilizes the zein/caseinate nanocomplex for the potential oral delivery of curcumin. *Molecules* (2019) 24(22):4061.
27. Chang C, Wang T, Hu Q, Zhou M, Xue J, Luo Y. Pectin coating improves physicochemical properties of caseinate/zein nanoparticles as oral delivery vehicles for curcumin. *Food Hydrocoll* (2017) 70:143-151.
28. Khan MA, Yue C, Fang Z, Hu S, Cheng H, Bakry AM, Liang L. Alginate/chitosan-coated zein nanoparticles for the delivery of resveratrol. *J Food Eng* (2019) 258:45-53.
29. Khan MA, Zhou C, Zheng P, Zhao M, Liang L. Improving physicochemical stability of quercetin-loaded hollow zein particles with chitosan/pectin complex coating. *Antioxidants* (2021) 10(9):1476.
